# Dr. Joseph Warren Hancock

**Published:** 1906-01

**Authors:** 


Dr. Joseph Warren Hancock, of Ellsworth, Wis., died at his home December 28, 1905, aged 57 years. He had served as County Judge of Pierce County (1885-1889) and was a member of the State Board of Health in 1894.

